# Home Ventilation

**Published:** 1916-05

**Authors:** 


**Home Ventilation.** Edward D. Rich, C. E., Public Health (Mich. State Board of Health), Jan. 1916. We omit the practical parts of this paper, since heating and ventilating systems can rarely be ordered by the medical attendant and their installation requires the services of a competent engineering architect. The following tables are of interest:

AIR POLLUTION TESTS

AIR DILUTION

| | Detrimental effect occurs in several hours. | ½ to 1 hour. |
|---|---|---|
| Iodine vapors | .00005 | .0003 |
| Chlorine or bromide vapors | .0001 | .0004 |
| Muriatic acid | .001 | .005 |
| Sulphuric acid | ...... | .005 |
| Sulphureted hydrogen | ...... | .02 |
| Ammonia | .01 | .03 |
| Carbonic oxide | .02 | .05 |
| Carbonic acid | 1.00 | 8.00 |
| Carbureted hydrogen | ...... | 6.56 gr. |

HEAT GIVEN OFF BY ILLUMINANTS

| Source. | Total B.T.U's Given off. |
|---|---|
| Gas—Fishtail burner | 313 |
| Gas—Argand burner | 198 |
| Gas—Welsbach burner | 32 |
| Petroleum | 198 |
| Incandescent lamp | 14 |
| Arc lamp | 2.5 |

CHANGE OF AIR NECESSARY

| | |
|---|---|
| Hospitals | 3,600 cu. ft. per person |
| Barracks and workshops | 3,000 cu. ft. per person |
| Schools | 2,400 cu. ft. per person |
| Churches, theaters and audience halls | 2,000 cu. ft. per seat |
| Office rooms | 1,800 cu. ft. |
| Toilet and bath rooms | 2,400 cu. ft. per fixture |
| Dining rooms | 1,800 cu. ft. per person |

For calculating the amount of air necessary, the following is given: S is the cubic feet of carbon dioxid per hour from each source, persons, lights, etc.; n the number of sources; a the allowable limit of carbon dioxid in 10,000 cubic feet of air; which should not exceed 7, 10 being the sanitary limit; A the number of cubic feet of air to be supplied per hour. A equals nS (or n S plus n′ S′ if there are different sources, as persons and gas lights) multiplied by 10,000 and divided by (a—4).

